# Intraperitoneally administered IgG from patients with amyotrophic lateral sclerosis or from an immune-mediated goat model increase the levels of TNF-α, IL-6, and IL-10 in the spinal cord and serum of mice

**DOI:** 10.1186/s12974-016-0586-7

**Published:** 2016-05-24

**Authors:** Izabella Obál, Gergely Klausz, Yvette Mándi, Mária Deli, László Siklós, József I. Engelhardt

**Affiliations:** Department of Neurology, University of Szeged, Szeged, Hungary; Second Department of Internal Medicine and Cardiology Center, University of Szeged, Szeged, Hungary; Department of Medical Microbiology and Immunobiology, University of Szeged, Szeged, Hungary; Laboratory of Molecular Neurobiology, Institute of Biophysics, Biological Research Center of the Hungarian Academy of Sciences, Szeged, Hungary

**Keywords:** ALS, IgG, Animal models, Spinal cord, Cytokines

## Abstract

**Background:**

Amyotrophic lateral sclerosis (ALS) is a neurodegenerative disease that involves the selective loss of the upper and lower motor neurons (MNs). Neuroinflammation has been implicated in the pathogenesis of the sporadic form of the disease. We earlier developed immune-mediated animal models of ALS and demonstrated humoral and cellular immune reactions in the nervous system and in the sera of patients and animals. The accumulation of immunoglobulin G (IgG), an elevated intracellular level of calcium, ultrastructural alterations in the MNs, and activation of the microglia were noted in the spinal cord of ALS patients. Similar alterations developed in mice inoculated intraperitoneally with IgG from ALS patients or from an immune-mediated goat model.

**Methods:**

We have now examined whether the intraperitoneal injection of mice with IgG from sporadic ALS patients or from immunized goats with the homogenate of the anterior horn of the bovine spinal cord is associated with changes in the pro-inflammatory (TNF-α and IL-6) and anti-inflammatory (IL-10) cytokines in the spinal cord and serum of the mice. The levels of cytokines were measured by ELISA.

**Results:**

Intraperitoneally administered IgG from the ALS patients induced subclinical signs of MN disease, while the injection of IgG from immunized goats resulted in a severe respiratory dysfunction and limb paralysis 24 h after the injections. Significantly increased levels of TNF-α and IL-10 were detected in the spinal cord of the mice injected with the human ALS IgG. The level of IL-6 increased primarily in the serum. The IgG from the immunized goats induced highly significant increases in the levels of all three cytokines in the serum and the spinal cord of mice.

**Conclusions:**

Our earlier experiments had proved that when ALS IgG or IgG from immune-mediated animal models was inoculated into mice, it was taken up in the MNs and had the ability to initiate damage in them. The pathological process was paralleled by microglia recruitment and activation in the spinal cord. The present experiment revealed that these forms of IgG cause significant increases in certain cytokine levels locally in the spinal cord and in the serum of the inoculated mice. These results suggest that IgG directed to the MNs may be an initial element in the damage to the MNs both in human ALS and in its immune-mediated animal models.

## Background

Amyotrophic lateral sclerosis (ALS) is a progressive neurodegenerative disease which primarily affects the upper and lower motor neurons (MNs) in the motor cortex, the brainstem, and the ventral horn of the spinal cord. Five to ten percent of ALS cases are considered to be familial (fALS), in which several genes have been found to be altered [1, 2]. The majority of ALS cases, however, are sporadic (sALS). Many hypotheses have been put forward to explain the selective degeneration of the MNs in ALS, including excitotoxicity [3–6], oxidative stress [7, 8], cytoskeletal abnormalities [9, 10], and the aggregation of abnormal proteins [2, 11]. These pathogenic mechanisms can be accounted in part by the genetic defects in fALS patients.

On the other hand, in sALS, the role of autoimmune mechanisms is increasingly emphasized [12–14]. The evidence indicates that the above processes are interrelated [6, 15]. Complex interactions between neurons and their non-neuronal neighbors have been documented in the pathological processes [13]. Non-neuronal cells contribute significantly to neuronal cell loss, mainly through neuroinflammatory processes [13, 14]. Glial cells and cells of the innate and acquired immune system are recruited and become activated. Reactive astrocytes in ALS can produce pro-inflammatory mediators, including interleukin-6 (IL-6) [16] and tumor necrosis factor-alpha (TNF-α) [17, 18].

Microglia accumulation and activation can be observed in close vicinity to MNs in the spinal cord of ALS patients [19–21]. The most potent antigen-presenting cell type, the dendritic cells, also appear in the affected area, and the conditions for local antigen presentation are therefore given in the central nervous system (CNS) tissue [19].

Moreover, both CD4+ and CD8+ T cell infiltrations can be detected in human autopsy spinal cord samples from ALS patients [21, 22]. The activation of the innate and humoral immunity was described also in ALS transgenic mice [23]. Signs of the activation of the humoral immune response can also be demonstrated. Immunoglobulin G (IgG) can be visualized postmortem in the spinal cord and cortical MNs of ALS patients [24]. IgG transferred intraperitoneally (ip) from sALS patients to mice is taken up by MNs [25], induces ultrastructural alterations and increases the calcium level in them [26–28], similarly to the ultrastructural alterations in the axon terminals from ALS patients in muscle biopsy samples [29].

Furthermore, sALS IgG that has accumulated in the cytoplasm of the MNs recruits activated microglia cells in the vicinity of the MNs [30]. It can also selectively induce the apoptosis of MNs via the caspase-3 pathway [31]. IgG produced by the immunization goats or guinea pigs with a ventral horn homogenate of the bovine spinal cord given ip to mice in another immune-mediated model of ALS evokes similar increases in intracellular calcium level and similar ultrastructural signs of MN degeneration to those induced by sALS IgG [32]. Moreover, it causes severe weakness and death of the recipients. A set of 20 antibodies characteristic of ALS directed to 20 human protein antigens were recently determined in the sera of ALS patients with a human protein array [33].

The immune-inflammatory reaction is associated with the production of neurotoxic cytokines and oxygen radicals in ALS. Experiments on transgenic mice overexpressing TNF-α, IL-6, and IL-3 in the CNS have demonstrated that the primary overexpression of these molecules without any external insult is sufficient to cause neurological deficits in vivo through either indirect or direct action on the MNs [34–36].

Ex vivo human data are also important as regards cytokines in sALS. Circulating levels of TNF-α and its soluble receptors are increased in the blood [37]. IL-6 [38, 39], transforming growth factor-beta (TGF-β) [40], IL-17, IL-23 [41], IL-12, and IL-15 [42] are also elevated in the serum and cerebrospinal fluid (CSF) of patients with ALS.

As opposed to the human diseases, cytokines can be determined directly in the CNS in animal models of MN diseases. Detectable TNF-α levels were reported in the CNS tissue of “mnd” mice (a model for a late-onset MN dysfunction) when the animals develop neurological symptoms [43]. Studies on SOD1 transgenic mice and rats demonstrated increased levels of TNF-α and TGF-β expression prior to the onset of a motor dysfunction [44–46]. Experiments on SOD1-mutant mice suggest that changes in cytokine production precede bulk protein oxidation and apoptosis gene expression [47].

The aim of the present study was to investigate cytokine production in mice inoculated ip with IgG from the sera of ALS patients or with anti-MN IgG from experimental immune-mediated animal models in order to establish whether IgG antibody taken up by the MNs is able to initiate changes in cytokine levels in the spinal cord and serum of the animals within 24 h.

Such detected changes would prove that, besides the local harmful effect on the MNs, anti-MN IgG can induce the production of certain cytokines in the spinal cord and in the serum of the animals, initiating an inflammatory reaction.

## Methods

### Patients and goats as sources of IgG and the preparation of IgG

Blood was obtained by phlebotomy from three sALS patients: two women and a man. The ages at diagnosis were 51, 55, and 59 years, and the duration of the disease from the first-noted symptoms was 1.5, 1, and 3 years. All three patients died, and autopsy samples were examined and partly reported on in this work. As controls, a gender and age-matched normal individual (woman), a woman with Parkinson’s disease and a man with multifocal motor neuropathy served as blood donors with their informed consent. The blood samples from the sALS patients were obtained within 1 month after establishment of the diagnosis. They had readily discernible upper and lower MN symptoms with bulbar involvement at the time of phlebotomy. The clinical diagnosis was established according to the El Escorial criteria [48] on the basis of medical history, physical examinations, electromyographic studies, exclusionary clinical, biochemical studies, and imaging techniques performed in order to rule out other diagnostic considerations. The autopsies demonstrated the pathological and histological signs of ALS and ruled out other diseases. The experiments were approved by the ethical committee of the University of Szeged. None of the patients received immunosuppressive treatment prior to serum collection. IgG was purified on an Avid/AL TM column (Bioprobe International, Inc. Tustin, CA, USA) according to the manufacturer’s instructions.

### Induction of experimental autoimmune gray matter disease (EAGMD) and production of anti-MN IgG

Three male goats were used to raise antibodies by active immunization. Preimmune sera had been obtained before the immunization, and the IgG was isolated from them as above. EAGMD was then induced by inoculation of the homogenate of the ventral horn of the bovine spinal cords [49]. Briefly, the goats were injected subcutaneously with a mixture of the spinal cord ventral horn homogenate and complete Freund’s adjuvant (1:1 ratio) at 10 sites over the back of the animals. One month later, the injections were repeated with a similar mixture, but containing incomplete Freund’s adjuvant. The animals were sacrificed under deep anesthesia 4 weeks later, when severe weakness had developed in the extremities and their blood was collected. IgG was purified according to the method listed above and was stored at −80 °C until used in injecting mice.

### Passive transfer experiments

Altogether, 62 mice were involved in the experiment. Twelve-week-old Balb/c mice were injected (two injections in 24 h) ip with 10 mg of one or other IgG sample. ALS group consisted of 3 + 2 + 2 mice inoculated with separate IgG samples from the three patients to measure the changes in the cytokine levels. Simultaneously, nine mice (3 + 3 + 3) were injected for histological examinations. Seven (3 + 2 + 2) mice were inoculated similarly with IgG from separate goats with EAGMD (goat group). Nine other mice (3 + 3 + 3) were inoculated with the same amount of IgG from the three goats for morphological examinations. Nine mice were injected with IgG from a healthy human individual, with IgG from the Parkinson disease patient or with IgG from multifocal motor neuropathy (three animals with each sample) and were regarded as negative human controls (control group). Their IgG were also inoculated into three mice from each individual for morphological tests. As further negative controls, three mice were inoculated with the vehicle of the IgG solution and nine mice with the preimmune goat IgGs; each goat IgG was injected into three mice (group 0). The mice in the group injected with anti-MN IgG were anesthetized near the terminal stage (24 h after the first injection), and blood samples were taken from the heart. The mice in the other groups were also sacrificed 24 h after the first injection. The spinal cords were removed and frozen immediately to −70 °C. Blood samples were centrifuged (20 min, 1600*g* at 4 °C), and the sera were stored at −70 °C until use. The spinal cord samples and sera were later processed for enzyme-linked immunosorbent assay (ELISA). All animal experiments were performed according to the appropriate institutional guidelines and governmental laws for animal protection.

### Determination of cytokine levels in serum and spinal cord samples of mice

ELISA was used to detect changes in the levels of all the pro-inflammatory TNF-α and IL-6 and anti-inflammatory (IL-10) cytokines in the passive transfer models of ALS in the mice injected ip with the IgG from the ALS patients (ALS group) and in the mice injected ip with the IgG from the goats with EAGMD (goat group). ELISA was also applied to measure the levels of the above cytokines in the mice inoculated with the IgG from the normal control human individual, from the Parkinson disease patient, or from the patient with multifocal motor neuropathy (control group). Finally, as control for the group of mice inoculated with the IgG from the EAGMD goats, the levels of the same cytokines were measured in mice inoculated with the IgG from the preimmune goat serum and with the vehicle of the IgG solution (group 0).

The immunosorbent assay kits of Biosource International, Inc. (Biosource, Camarillo, CA, USA) were used for quantitative determination of the abovementioned cytokines in the serum and spinal cord samples of mice. Antigen retrieval in spinal cord samples was enhanced by means of homogenization with ultrasound for 20 s. The protein contents of the samples were determined by using the bicinchoninic acid assay (Pierce TM Thermo Scientific TM, Rockford, IL, USA). The protein contents of the spinal cord samples were adjusted to 1 mg/ml. The TNF-α, IL-6, and IL-10 levels in the homogenates were determined with the ELISA kits according to the manufacturer’s instructions. Serum and spinal cord samples and appropriate standards were pipetted into wells coated with either a polyclonal antibody specific for mouse (m)-TNF-α, a monoclonal antibody specific for (m)-IL-6, or a monoclonal antibody specific for (m)-IL-10. After incubation, biotinylated monoclonal secondary antibodies were added, followed by streptavidin-peroxidase, and the incubation was repeated. After incubation and washing, the bound cytokines were visualized by developing the peroxidase reaction through the addition of H_2_O_2_, and the absorbency of each well was determined by means of an ELISA reader. Sera from the immunized goats (EAGMD) and ALS patients were also used as controls in order to test for antibody cross-reactivity during the ELISA with human and goat cytokines.

### Statistical analysis of the data

**Fig. 1:**
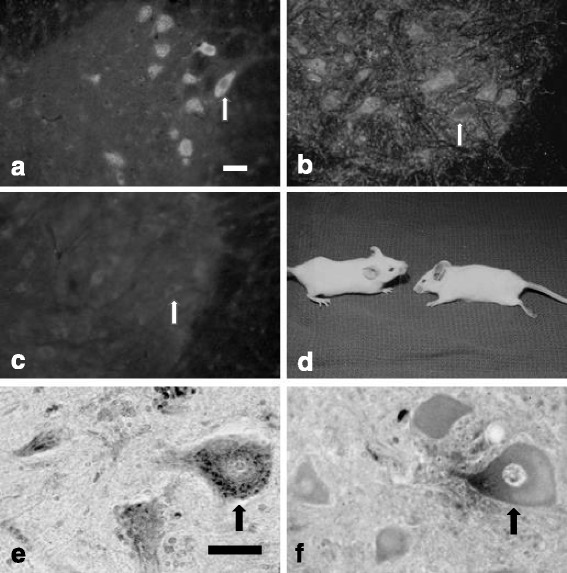
Photographs of the animals and histological pictures of the accumulation of IgG in the spinal MNs of inoculated mice and from human autopsy samples. **a** The accumulation of ip administered ALS IgG in the spinal MNs of mice (one is indicated by *an arrow*) detected by direct immunohistochemistry with FITC-labeled antibody reacting with human IgG. Fluorescence picture. *The bar* indicates 30 μm for each picture: (**a**–**c**). **b** The accumulation in the spinal MN of a mouse of ip inoculated IgG from a goat immunized with the homogenate of the ventral horn of the bovine spinal cord, visualized with FITC-labeled anti-goat IgG. Direct immunohistochemistry. Both the MNs and the cell processes contain goat IgG, and the external membrane of the MNs is immunostained (indicated by *an arrow*). **c** Slight IgG immunoreactivity in the spinal MNs of mice (one is indicated by *an arrow*) after the ip inoculation of normal human IgG. **d** The mouse on the *left* was inoculated with IgG obtained from the deceased sALS patient whose spinal cord is shown in (**e**). The body posture of the animal appears unusual, reflecting some decrease in muscle strength, but obvious paresis did not develop. The mouse on the *right* was completely paralyzed after the ip injection of IgG from a goat with EAGMD. **e** Immunohistochemical picture of the ventral horn MNs from the lumbar region of the spinal cord of a patient who died of ALS. IgG accumulation can be detected in granular form in the cytoplasm of the MNs with peroxidase-labeled anti-human IgG as a dark reaction product. (One is indicated by the *arrow*.) The bar represents 60 μm. **f** The spinal MNs from autopsy sample of the lumbar spinal cord of a patient without CNS disease exhibits minimal immunostaining for human IgG with the same immunohistochemical reaction as in (**e**) (*arrow*)

**Fig. 2:**
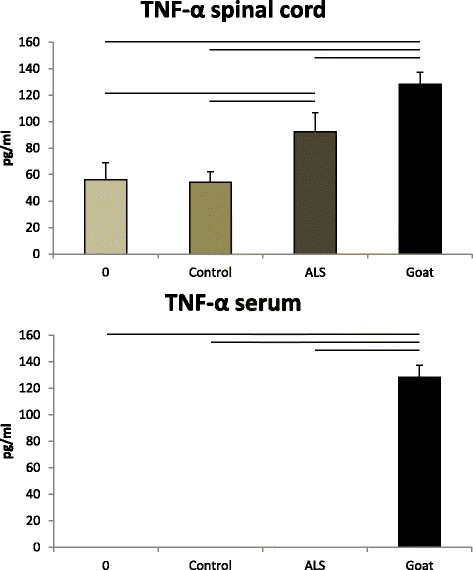
The levels of TNF-α in the spinal cord and serum of the inoculated mice. *Upper*. Certain levels of TNF-α were noted in the spinal cord of the mice in group 0 and in control group. However, the level of TNF-α in the spinal cord of the mice injected ip with 10 mg of the IgG from the ALS patient (ALS group) was significantly higher than in the mice inoculated with the IgG from the human normal and disease controls (control group), and in the mice inoculated with the vehicle (without IgG) or with preimmune goat IgG (group 0). The level of TNF-α in the spinal cord of the mice inoculated ip with the IgG from the goats immunized with the homogenate of the ventral horn of the bovine spinal cord (goat group) was significantly higher than in the other three groups. The significance of the differences is indicated by the *horizontal lines. Lower*. No TNF-α was detected in the serum of the animals in group 0, control group, or ALS group, while the serum of the mice in the goat group contained a high level of TNF-α. The data indicate that the IgG from ALS patients raised the level of TNF-α only in the spinal cord of the injected mice. In every figure, the *columns* denote means and the *error bars* the SD

**Fig. 3:**
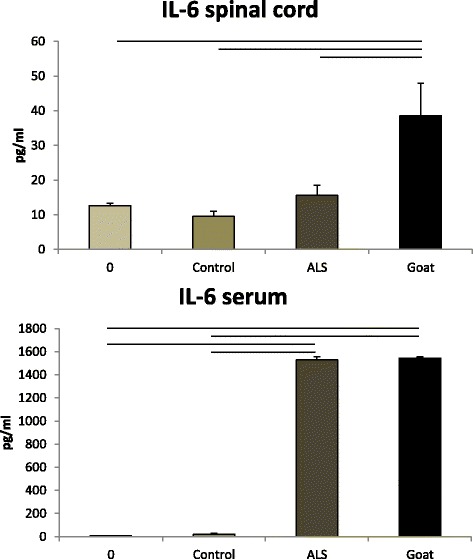
The levels of IL-6 in the spinal cord and the serum of the inoculated mice. *Upper*. There were demonstrable levels of IL-6 in the spinal cord in group 0 and in control group. The average level of IL-6 was higher in the spinal cord of the mice inoculated ip with 10 mg of ALS IgG, but the difference was not statistically significant. The average level of IL-6 was statistically significantly elevated in the mice in the goat group (inoculated with IgG from the serum of the immunized goats). *Lower*. Mouse IL-6 was not detected in the serum of the mice in group 0 (injected with only vehicle or preimmune goat serum), and only traces of IL-6 were found in the serum of the mice treated with normal human or disease control IgG (control group). The treatment with human ALS IgG (ALS group) or with IgG from the immunized goats raised the IL-6 level extremely in the serum of the inoculated mice

One-way ANOVA followed by the Student-Newman-Keuls test was used for statistical comparison of the data from four groups of mice (Figs. 1, 2, and 3): the effects of the IgG from the ALS patients (ALS group) and the IgG from the paralyzed goats immunized with the homogenate of the ventral horn of the bovine spinal cord (goat group) were compared with those on the appropriate control groups: inoculated with IgG from the healthy human control, from the patient with Parkinson’s disease or from the multifocal motor neuropathy case (control group), and that on mice injected with vehicle or preimmune goat serum (group 0).

### Histological processing

The mice injected with ALS IgG or anti-MN IgG and the control mice were perfused through the heart under deep flurane anesthesia with 100 mmol/l phosphate-buffered saline (PBS) followed by 4 % neutral paraformaldehyde. The spinal cords were removed and postfixed in the same fixative by immersion, then kept in 30 % sucrose containing PBS to prevent the formation of freezing artifacts. The pieces of the lumbar spinal cords were frozen in 2-methyl-butane and 20-μm-thick cross-sections were cut in a cryostat. After rinsing in PBS, the non-specific IgG binding sites were blocked by floating in 5 % rabbit serum (Sigma-Aldrich) containing PBS. The sections from animals inoculated with goat IgG were then floated on a rocker for 3 h in a 1:400 dilution of FITC-labeled anti-goat IgG (whole molecule) produced in rabbit (Sigma-Aldrich) containing 3 % normal rabbit serum. In sections from animals injected with human IgG, the specific antibody-binding sites were blocked in the same way and were incubated with a 1:400 dilution of anti-human IgG F(ab’)_2_ highly cross-adsorbed FITC-labeled antibody produced in rabbit (Sigma-Aldrich) in the same way as above. After rinsing, the sections were placed on glass slides, coverslipped, and examined in a Nikon Optiphot fluorescence microscope. Samples from control animals served as controls. As method control, the fluorescence antibody dilutions were incubated with normal human serum and/or normal goat serum, the precipitates were then removed by centrifugation and the supernatants were used for immunostaining.

One-centimeter long pieces of the lumbar spinal cords obtained from the autopsies on the three ALS patients and controls were fixed by immersion in 5 % paraformaldehyde and were treated for sectioning in a cryostat as above. Twenty-micrometer sections were cut, rinsed, blocked by floating in 5 % normal rabbit serum, and then incubated as above with a 1:400 dilution of peroxidase-labeled anti-human IgG (whole molecule) produced in rabbit (Sigma-Aldrich). After rinsing, the sections were transferred to Fisherbrand Superfrost^™^ Plus microscope slides (Fisher Scientific). The immunoreaction was developed by using an enhanced diaminobenzidine substrate kit for 15 min (Pierce, Rockford, IL, USA). The sections were washed in distilled water, dehydrated in a series of ethanol, cleared in xylene, coverslipped, and examined in a Nikon Optiphot light microscope. Samples from the control human spinal cords from autopsy materials from individuals not suffering from CNS diseases served as controls. As method control, the peroxidase-labeled antibody dilutions were incubated with normal human serum, the precipitates were next removed by centrifugation and the supernatants were used for immunostaining.

## Results

### Behavior of the injected mice

Each of the mice injected ip with the IgG from the ALS patients developed slightly sluggish movements generally. This slight, but noticeable deficit is comparable with the altered ultrastructure and increased calcium content in the MNs of mice inoculated with IgG from ALS patients described previously [26]. Nevertheless, obvious paralysis was not noted. As opposed to this, the mice injected with the IgG from the EAGMD goats with limb paralysis developed severe weakness in the limbs and could not walk at all at the end of the 24-h observational period (Fig. 1d). The mice injected with the preimmune IgG from the same goats, or with the IgG from a normal human individual, from the Parkinson disease patient or from the patient with multifocal motor neuropathy, or with vehicle did not appear to exhibit any change in movement or behavior.

### Immunohistochemical examinations of the lumbar spinal cord of the mice inoculated with the IgG from the sALS patient or from the goats with EAGMD and the normal control IgG

FITC-labeled anti-human IgG revealed the accumulation of the human IgG in the ventral horn MNs of the mice inoculated ip with the IgG from the sALS patients. The staining pattern was cytoplasmic, leaving the nucleus unstained (Fig. 1a). In certain MNs, the staining was enhanced in a granular pattern. The IgG of the three ALS patients all gave similar MN staining patterns. Cells other than the ventral horn MNs did not seem to be stained. The lumbar spinal cord of the mice injected ip with the IgG from the immunized goats exhibited a different staining pattern with FITC-labeled anti-goat IgG. The goat IgG similarly accumulated in the MNs of the ventral horn, but the external membranes and processes of the cells were also heavily stained (Fig. 1b). The IgG from all three goats gave similar staining patterns. Only traces of IgG were detected with the same methods in the mice inoculated ip with the preimmune IgG from the goats or the IgG from the human controls (Fig. 1c).

### Changes in cytokines in the spinal cord and serum of the inoculated mice

The levels of TNF-α in the spinal cord of the animals inoculated with the preimmune goat IgG or with vehicle (group 0) were similar to those in the animals inoculated with the IgG from the normal human individual, from the Parkinson patient or from the patient with multifocal motor neuropathy (control group): 56 ± 12.78 pg/ml and 54.2 ± 8.12 pg/ml (means ± SD). The level of TNF-α in the animals injected with ALS IgG (ALS group) rose to 92.4 ± 14.38 pg/ml, and that in the mice injected with EAGMD IgG (goat group) to 128.4 ± 8.76 pg/ml. Meanwhile, there was no measurable TNF-α in the serum of the mice in group 0, control group, or ALS group (inoculation with the IgG from the ALS patients). The IgG of the goats with EAGMD induced a significant increase in the level of TNF-α in the serum of the inoculated mice, albeit it was less than that in the spinal cord (Fig. 2).

The spinal cord level of IL-6 was 12.6 ± 0.658 pg/ml in group 0 and 9.5 ± 1.53 pg/ml in control group. It was higher, but not statistically significant in the ALS-IgG treated animals (15.6 ± 2.878 pg/ml). In contrast, the IgG from the EAGMD goats raised the IL-6 level significantly in the spinal cord of the injected mice (38.6 ± 9.378 pg/ml). The IL-6 level was increased significantly in the serum of the mice injected either with ALS IgG or with the IgG from the immunized goats (1528.34 ± 29.36 and 1543.62 ± 12.36 pg/ml). The serum of group 0 did not reveal IL-6, while that group of the control contained only traces of mouse IL-6 (Fig. 3).

**Fig. 4:**
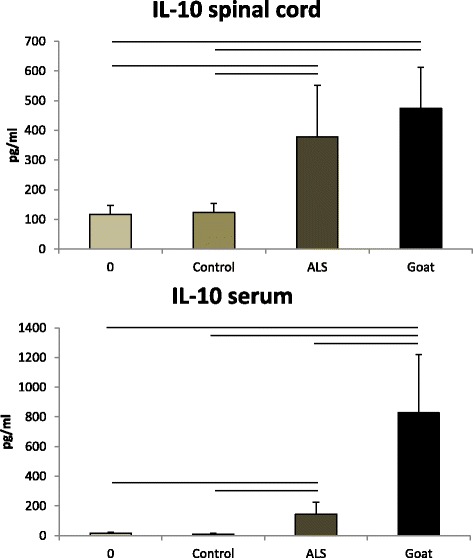
The levels of IL-10 in the spinal cord and serum of the inoculated mice. *Upper*. The levels of IL-10 in the spinal cord of the mice in group 0 and control group were almost the same. The spinal cord of the mice inoculated with the IgG from the ALS patients (ALS group) or from the immunized goats with EAGMD (goat group) contained highly increased levels of IL-10. *Lower*. IL-10 was not detected in the serum of the mice in group 0 or control group. The serum of the animals in the ALS group and even more so in the goat group contained significantly higher levels of IL-10

IL-10 was also demonstrable in the spinal cord of group 0 (117 ± 29.4 pg/ml) and control (123 ± 30.6 pg/ml). On the other hand, there was a 3–4-fold increase in the spinal cord after treatment with the IgG from the ALS patients (378 ± 173.6 pg/ml) or the immunized goats (473.8 ± 138 pg/ml). The serum in group 0 and control group contained only traces of mouse IL-10. The serum of the mice after inoculation with the ALS IgG contained 144.28 ± 82.12 pg/ml IL-10, which was a little less than one third of the spinal cord level. The level of IL-10 in the serum of the mice treated with the IgG from the immunized goats was 827.86 ± 390 pg/ml, which is higher than that in the spinal cord (473.8 ± 138 pg/ml) (Fig. 4). TNF-α was increased significantly in the spinal cord of the mice injected with the sALS IgG or the IgG from the immunized goats with EAGMD [ANOVA *F*(3,22) = 62.5, *p* < 0.0001]. However, an increase in the serum TNF-α level occurred only in the mice injected with the IgG from the goats with EAGMD [ANOVA *F*(3,22) = 13.4, *p* < 0.0001]. The other possible neurotoxic cytokine, IL-6, was increased significantly in the spinal cord of the mice injected with the IgG from the goats with EAGMD, but not after the sALS IgG [ANOVA *F*(3,22) = 44.0, *p* < 0.0001. Highly significant increases in IL-6 levels were noted in the serum of the mice injected with either sALS IgG or the IgG from the goats with EAGMD [ANOVA *F*(3,22) = 16996.4, *p* < 0.0001].

The levels of the anti-inflammatory cytokine IL-10 in the spinal cord of the mice injected with the IgG from the sALS patients or with the IgG from the goats with EAGMD were also elevated [*F*(3,22) = 15.4, *p* < 0.0001], and they were also enhanced in the serum [ANOVA *F*(3,22) = 23.5, *p* < 0.0001].

None of the inoculated goat and human IgG-s contained mouse TNF-α, IL-6, or IL-10.

### Immunohistochemical examinations of the lumbar spinal cord of the deceased ALS patients and age-matched controls who died of non-neurological diseases

Immunostaining for IgG with peroxidase-labeled antibodies demonstrated that most of the MNs in the lumbar spinal cord of all three sALS patients contained IgG in the cytoplasm. The dark, mostly granular reaction product accumulated mainly in the rough endoplasmic reticulum, indicating that this is the main target for antibody binding (Fig. 1e). The MNs in the ventral horns of the lumbar spinal cord exhibited no or very little dark immunostaining and not in a granular manner, indicating that IgG did not accumulate in the cells (Fig. 1f).

## Discussion

Our main purpose was to investigate whether the uptake of ALS IgG or anti-motoneuron IgG can initiate a further event in the supposed inflammatory reaction in the spinal cord of the injected mice at the very beginning of the injury of the motor neurons. We expect that this event is the initiation of the production of pro-inflammatory cytokines in the spinal cord after the intra-motoneuronal accumulation of ALS specific IgG. We already proved that macrophage chemoattractant protein-1 is highly elevated in the spinal cord of ALS patients (Ref. 19). Therefore, we investigated the appearance of the most important pro- and anti-inflammatory cytokines, such as TNF-α, IL-6, and IL-10. The reasons why we chose these three cytokines are the following: TNF-α and IL-6 play an important role in orchestrating and influencing the induction of the cytokine cascade, initiating an inflammatory reaction, and affecting the production of further cytokines. We also wanted to know whether any sign of anti-inflammatory reaction can also be shown, because in these acute passive transfer experiments, ALS IgG does not cause the death of spinal MNs, only the initial morphological and electrophysiological alterations of the degeneration were noted. That was the reasoning behind choosing IL-10.

The inoculation of ALS and anti-MN IgG from EAGMD goats induced different changes in the levels of cytokines in the serum and in the spinal cord in the various mouse groups. The spinal cords of the mice in group 0 and control group (not treated with the ALS IgG or the IgG from the goats with EAGMD) contained more TNF-α as compared with the serum. The administration of the sALS IgG increased the level of TNF-α in the spinal cord significantly, but not the serum content. The IgG from the paralyzed goats elevated the level of TNF-α in the spinal cord of the injected mice more (these mice also became paralyzed), but the level of TNF-α in the serum reached only about half of the concentration measured in the spinal cord. These results indicate that the primary site of the elevation of TNF-α caused by the sALS IgG or the IgG from the immunized goats is the spinal cord. In both experimental paradigms, IgG is known to accumulate in the spinal cord MNs [25, 32], and signaling for the enhancement of TNF-α is presumably initiated by the IgG accumulation there, though the pathway is unknown. TNF-α is mainly produced by the microglia, but the astrocytes and injured neurons can also secrete this cytokine [18, 50]. Previous passive transfer experiments with IgG from sALS given ip to mice revealed that microglia were recruited in the vicinity of MNs filled with sALS IgG [30]. TNF-α has been shown to potentiate glutamate-mediated cytotoxicity by two complementary mechanisms: indirectly by inhibiting glutamate transport to the astrocytes and directly by rapidly triggering the surface expression of calcium-permeable AMPA receptors and NMDA receptors, while decreasing inhibitory GABA_A_ receptors on the neurons [6]. TNF-α additionally induces oxidative stress, and is therefore able to interconnect all of the implicated pathomechanisms in ALS [6].

The IL-6 level was significantly increased in the spinal cord of the mice inoculated with the IgG from the immunized goats (approximately three times higher than in the other groups), whereas it was approximately 40 times higher in the serum than in the spinal cord after treatment with the IgG from the goats with EAGMD and approximately 100 times higher after the inoculation of the sALS IgG (Fig. 3). These results suggest that the increase in the level of IL-6 is mostly induced by the general and systemic effects of sALS IgG and IgG from the goats with EAGMD on the whole organism, and not primarily in the spinal cord.

Astroglia isolated from postmortem tissue of both fALS and sALS patients have been shown to be toxic to MNs [51]. Reactive astrocytes can secrete increased amounts of IL-6 and TNF-α, and the TGF-β level has been found to be elevated in the CSF of sALS patients; the observed elevations may originate in part from these cells besides activated T lymphocytes. Another source of IL-6 is the classically activated microglia. Microglia belonging in the M1 group are neurotoxic and produce pro-inflammatory cytokines (TNF-α, IL-1β, IL-6, IL-12, and IL-23) and reactive oxygen species [52]. Moreover, during the rapidly progressing phase of the disease process, cytotoxic CD4+ and CD25− T lymphocytes (Teffs) proliferate in ALS. This corroborates findings on ALS mice [53]. Another subset of T cells, Th17, that produce IL-17 has also been suggested to be crucial in MN destruction in ALS [54]. IL-6, a secondary cytokine, is involved in the differentiation, maintenance, and stabilization of Th17 cells [55] and Th2 lymphocytes [56]. Th2 cell differentiation is induced through the expression of IL-4 and the suppression of γ-IFN [57, 58]. IL-6 also inhibits the differentiation of naive CD4+ T cells into Treg lymphocytes [56]. The suppression of Treg cell differentiation and the induction of Th17 lymphocytes are mediated by IL-6 trans-signaling, since the IL-6 soluble receptor is necessary for these effects [59]. The lack of lymphocyte recruitment previously demonstrated in the ALS IgG-injected group [30] is supported by the fact that only a slight, non-significant elevation was observed in the level of spinal cord IL-6 in our present experiments. As a secondary cytokine responsible for enhancing the innate immune response and promoting the development of Th2 and Th17 lymphocytes, IL-6 seems to be important in this process. Because of the insufficient signals during the short time scale of passive transfer models, lymphocytes might not become activated, whereas in ALS patients, where IL-6 and other secondary cytokines have been detected in the CSF and serum, the immune system can be activated to its full extent. The elevation of IL-6 observed in the serum of the sALS-IgG-injected mice suggests that the involvement of further components of the immune response is encouraged systemically.

Endothelial cells might also contribute to the high levels of IL-6 observed in the serum. Further involvement of the surrounding cells can occur especially in the case of the mice injected with the IgG from the immunized goats, when the animals are in a state of acute distress, with progressive limb paralysis and insufficient respiration due to the weakness of the striated muscles. As in infection-induced inflammation, the endothelial cells may secrete increased amounts of IL-6, which can exaggerate inflammation through trans-signaling mechanisms [56].

IL-10 was demonstrable in the spinal cord in all the groups of mice, even in group 0 and control group. The level of IL-10 was elevated significantly in the spinal cord of the sALS IgG-inoculated mice, but the increase in the serum did not attain statistical significance. In contrast, concordant and significant elevations were observed in both the spinal cord and the serum of the mice injected with the IgG from the immunized goats. IL-10 is an anti-inflammatory cytokine, and the fact that ALS IgG induces a comparably higher elevation in the spinal cord, therefore suggests that, although there is a simultaneous anti-inflammatory reaction, it primarily starts in the spinal cord. In the mice inoculated with the IgG from the immunized goats, the IL-10 levels in both the spinal cord and the serum were higher than in the ALS IgG-injected mice, but the serum contained more IL-10 than the spinal cord. The simultaneous production of the anti-inflammatory cytokine IL-10 is presumed to hinder inflammation here too. However, this mechanism seems to be more effective in the ALS IgG-inoculated mice, where IL-10 is present in a higher level in the spinal cord than in the serum. This may be one of the reasons why ALS IgG, despite the initiated inflammation, does not lead to the paralysis of the injected mice. Another possible reason might be that the inoculated IgG binds to different antigens. ALS IgG has been shown to be bound only to intracellular antigens [25], whereas IgG from EAMND is bound to both intracellular and external cell membrane antigens [49].

The levels of all three examined cytokines were elevated by the ip injection of the IgG from the ALS patients or from an autoimmune animal model of ALS (EAGMD). We established earlier that the specific IgG antibodies characteristic of the serum of ALS patients are directed against human proteins mostly found in the MNs [33]. However, it is not known which are responsible for the alterations of the ultrastructure, calcium homeostasis, physiological abnormalities of the MNs, and the increased levels of cytokines. The serum of immunized goats was not subjected to such an evaluation. On the other hand, all of the alterations mentioned above developed after the ip administration of IgG to mice, similarly to the effects of ALS IgG. This suggests that the primary insult to the MNs may also attribute to the possible effect of autoimmune IgG.

The innate immune response that takes place in the CNS during acute systemic infection and acute trauma is unlikely to be detrimental to the brain. On a short time scale, neurotrophic factors and other molecules are produced that have important roles in brain homeostasis, neuroprotection, and repair. However, when the infection or insult becomes chronic or severe, e.g., in neurodegeneration such as ALS, the delicate balance between the pro- and anti-inflammatory mechanisms is disrupted. In the pathology of ALS, as the disease progresses, the astrocytes and microglia transform from neuroprotective (M2) to neurotoxic (M1), humoral antibodies are deposited, and activated T cells accumulate, as do “AbA cells”, aberrant astrocytes in SOD1 mutant rats [60]. M2 microglia secrete anti-inflammatory cytokines (IL-10) and neurotrophins (NGF, BDNF and IGF-1). In fALS mouse models, an M2 microglia response predominates at the beginning of the disease, followed by M1 neurotoxic signals toward the terminal stages [61]. The published data indicate that the microglia induces MN death via pro-inflammatory mechanisms through NF-kB signaling [62].

## Conclusions

In previous, well-characterized, immune-mediated models of sALS [63], and specific immunological reactions were detected in and around the perikarya of the spinal cord MNs and also in the axon terminals. These findings correspond with data obtained from ALS autopsy and biopsy samples [19, 24, 29]. Cytokine production, presumably following these inflammatory responses, has not been investigated in immune-mediated, passive transfer models. Our present experiments revealed characteristic changes in the levels of pro-inflammatory cytokines TNF-α, IL-6, and anti-inflammatory cytokine IL-10 both in the spinal cord and in the serum of mice injected ip with the IgG from the sALS patients or IgG from goats immunized with the homogenate of the ventral horn of the bovine spinal cord. Additional data emerged concerning the pathomechanism of MN injury simply the uptake of autoimmune IgG directed to the constituents of MNs is sufficient to initiate an autoimmune reaction causing ultrastructural alterations, dysregulated calcium homeostasis of the MNs, activation and recruitment of the microglia in the vicinity of the MNs, and the elevation of pro- and anti-inflammatory cytokines.

## Abbreviations

ALS, amyotrophic lateral sclerosis; BDNF, brain-derived neurotrophic factor; CNS, central nervous system; CSF, cerebrospinal fluid; EAGMD, experimental autoimmune gray matter disease; ELISA, enzyme-linked immunosorbent assay; fALS, familial amyotrophic lateral sclerosis; FITC, fluorescein isothiocyanate; IFN-γ, nterferon gamma; IgG, immunoglobulin G; IL, interleukin; MN, motor neuron; NF-kB, nuclear factor kappaB; NGF, nerve growth factor; PBS, phosphate-buffered saline; sALS, sporadic amyotrophic lateral sclerosis; SD, standard deviation; TGF-β, transforming growth factor beta; TNF-α, tumor necrosis factor alpha.
